# Targeting toll-like receptor 7/8 for immunotherapy: recent advances and prospectives

**DOI:** 10.1186/s40364-022-00436-7

**Published:** 2022-12-07

**Authors:** Hao Sun, Yingmei Li, Peng Zhang, Haizhou Xing, Song Zhao, Yongping Song, Dingming Wan, Jifeng Yu

**Affiliations:** 1 Department of Radiotherapy, the First Affiliated Hospital of Zhengzhou University, Zhengzhou, 450052 Henan China; 2 Department of Hematology, the First Affiliated Hospital of Zhengzhou University, Zhengzhou, 450052 Henan China; 3 Department of Thoracic Surgery, the First Affiliated Hospital of Zhengzhou University, Zhengzhou, 450052 Henan China; 4 Henan International Joint Laboratory of Nuclear Protein Gene Regulation, Henan University College of Medicine, Kaifeng, 475004 Henan China

**Keywords:** Toll-like receptors (TLRs), Agonist, Immune checkpoint pathway, cancer immunotherapy, Chronic hepatitis B, Clinical trial

## Abstract

Toll-like receptors (TLRs) are a large family of proteins that are expressed in immune cells and various tumor cells. TLR7/8 are located in the intracellular endosomes, participate in tumor immune surveillance and play different roles in tumor growth. Activation of TLRs 7 and 8 triggers induction of a Th1 type innate immune response in the highly sophisticated process of innate immunity signaling with the recent research advances involving the small molecule activation of TLR 7 and 8. The wide range of expression and clinical significance of TLR7/TLR8 in different kinds of cancers have been extensively explored. TLR7/TLR8 can be used as novel diagnostic biomarkers, progression and prognostic indicators, and immunotherapeutic targets for various tumors. Although the mechanism of action of TLR7/8 in cancer immunotherapy is still incomplete, TLRs on T cells are involved in the regulation of T cell function and serve as co-stimulatory molecules and activate T cell immunity. TLR agonists can activate T cell-mediated antitumor responses with both innate and adaptive immune responses to improve tumor therapy. Recently, novel drugs of TLR7 or TLR8 agonists with different scaffolds have been developed. These agonists lead to the induction of certain cytokines and chemokines that can be applied to the treatment of some diseases and can be used as good adjutants for vaccines. Furthermore, TLR7/8 agonists as potential therapeutics for tumor-targeted immunotherapy have been developed. In this review, we summarize the recent advances in the development of immunotherapy strategies targeting TLR7/8 in patients with various cancers and chronic hepatitis B.

## Introduction

Toll-like receptors (TLRs) are a large family of proteins and a class of pattern recognition receptors, which are not only expressed in immune cells, but also in various tumor cells. As the key components, TLRs are evolutionarily conserved innate immune molecules that play an important part in the innate immune system and promote adaptive immune responses as a bridge between innate immunity and adaptive immunity. Different TLRs are expressed differently in different target cells and play different functions by activating different immune cascades [1]. There are ten kinds of TLRs in the human (TLR1-TLR10) and 13 kinds of TLRs in mice. TLR1, TLR2, TLR4, TLR5, TLR6, TLR10, and TLR11 are receptors located on the surface of the cell membrane that recognize extracellular components of pathogens, and TLR3, TLR7, TLR8, TLR9, and TLR13 are located on endosomes where they recognize foreign nucleic acids [2, 3]. As a member of the TLR family with less known functions, TLR13 can participate in the immune and inflammatory reactions for recognizing the conserved sequence of 23S rRNA in bacteria and induce immune response in mice [4, 5]. TLRs can recognize pathogen-related and structurally conserved molecules like single-stranded (ss) or double-stranded (ds) RNAs or DNAs, lipoproteins and lipopolysaccharides derived from microbes, and then activate immune cell responses to all externally attacking microbiota [2]. They participate in tumor immune surveillance and play different roles in tumor growth [6]. Activation of TLR4/9-COX2 signaling was involved in the metastasis of hepatocellular carcinoma. Inhibition of TLR4/9-COX2 signaling abrogated the neutrophils to form extracellular traps (NETs)-aroused metastatic potential [7].

The activation of TLRs downstream signal mainly depends on two types of transcription factors: NF-κB and interference regulatory factors (IRFs), which mainly induce the production of pro-inflammatory cytokines. In addition, it can induce the production of type I interferon (IFN) [2]. Activation of TLR7 and TLR8 triggers induction of a Th1-type innate immune response. The emergence of new structural and small molecule information generated in the last decade has contributed enormously to our understanding of this highly sophisticated process of innate immunity signaling with the recent developments in the small molecule activation of TLR7 and TLR8 [8].

Meanwhile, many studies have revealed that the expression levels of TLR7 and TLR8 are altered in some autoimmune diseases, such as arthritis, cancers [9–13], or in antiviral regimes, including coronavirus and human immunodeficiency virus(HIV) prevention [14]. TLR agonists have potential therapeutic prospects and are one of the research hotspots in the field of immunotherapy. Thus, novel drugs of TLR7 and TLR8 agonists with different scaffolds have been developed. These agonists can induce certain cytokines and chemokines that can be used as good adjuvants for vaccines in the treatment of some diseases. Furthermore, TLR7/8 agonists as potential therapeutics for tumor-targeted immunotherapy have been developed [8, 15, 16].

In this review, we summarize the frontiers of TLR 7 and 8 related research, especially, in the field of new drug development of TLR agonists for cancer immunotherapy.

## TLR family and its expression patterns

**Fig. 1:**
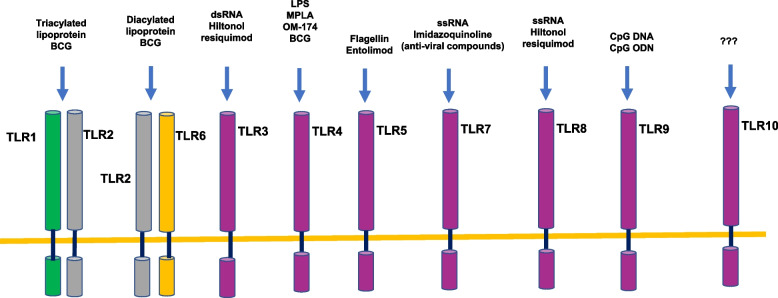
TLRs and their ligands. TLR1–7 and TLR9 have been characterized to recognize microbial components. TLR3 is essential for the recognition of microbial lipopeptides. TLR1 and TL6 associate with TLR2, and discriminate subtle differences between triacyl- and diacyl lipopeptides, respectively. TLR4 recognizes LPS. TLR9 is the CpG DNA receptor, whereas TLR3 is implicated in the recognition of viral dsRNA. TLR5 is a receptor for flagellin. TLR10 is the latest human TLR to be discovered and its function and ligands are still unclear

A typical TLR is a single-spanning receptor consisting of three domains: an extracellular domain (ECD) for the recognition of pathogen-associated molecular patterns (PAMPs), a transmembrane domain (TMD), and an intracellular Toll-interleukin 1 receptor (TIR) domain for initiating downstream signaling [17]. Each of these receptors has a unique antigen-recognizing domain [18]. TLR3, TLR7, TLR8, and TLR9 act as sensors of nucleic acids. Specifically, TLR3 recognizes viral dsRNA, and TLR9 senses unmethylated cytosine phosphate guanosine (CpG) containing DNA, whereas TLR7 and TLR8 function as viral ssRNA sensors [19]. TLR4 identifies bacterial lipopolysaccharide (LPS) found in gram-negative bacteria, whereas TLR5 recognizes flagellin, and TLR9 subfamily members (TLR7, TLR8, and TLR9) recognize microbial DNA and RNA [18]. TLR10 is the latest discovered human TLR, and its ligands are still unknown (Fig. 1). However, TLR10 is the only known member of the TLR family that can elicit an anti-inflammatory effect [3]. The intracellular TLRs (TLR3, TLR7, TLR8, and TLR9 in human) can detect viral and bacterial nucleic acids, playing an important role in host immune response [20–25] and potentially in the treatment of cancer [26].

In contrast, extracellular TLRs, such as TLR1, TLR2, TLR4, TLR5, and TLR6, can be located at the plasma membrane where they recognize macromolecules exposed on the surface of pathogens [20–25]. Emerging evidence has suggested that dysfunction of TLRs has been correlated with inflammation associated with tumorigenesis (carcinogenesis), such as esophageal cancer [20] and other gastrointestinal tract cancers [27, 28]. Specifically, expression of TLR3, TLR4, TLR5, and TLR9 has been suggested as a potential mediator of the progression from reflux disorders to esophageal adenocarcinoma [29] Meanwhile, the increased expression levels of TLR3, TLR4, and TLR9 have been observed in esophageal squamous cell carcinoma (SCC) associated with lymphatic metastasis, with increased expression of TLR7 and TLR9 associating with advanced disease [30]. TLRs’ expression may be used as a valuable diagnostic or prognostic factor for esophageal cancer [20] .

TLR7, TLR8, and TLR9 display similarities in structure and endosomal localization, and natural agonists composed of nucleic acids, such as ssRNA or DNA with CpG motifs, activate the innate immune cells through these TLRs. The modulation of TLR7 and TLR8 responses is independent of CpG motifs or the nature of the oligodeoxynucleotides (ODNs) backbone structure, and the crosstalk between ODNs, IRMs, and TLR7 and TLR8 may be used for different clinical implications, including tumor therapy [31].

Although TLR7 and TLR8 show a high degree of sequence homology and similar structure, their biological responses to small molecule binding are very different. A recent study on molecular dynamics simulations reveals the selectivity mechanism of structurally similar agonists to TLR7 and TLR8, each with their own specific adapter proteins [32, 33]. While TLR7 is mainly expressed in antigen-presenting cells (APCs), such as plasmacytoid dendritic cells (pDCs) and B-cells, TLR8 is primarily expressed in myeloid cells, such as monocytes, macrophages, and myeloid dendritic cells [33]. Furthermore, TLR8 is considered biologically inactive in mice because they are unresponsive to imidazoquinolines (TLR7/8 agonists) when TLR7 expression has been knocked down. Hence, murine TLR8 cannot be triggered by TLR7/8 agonists [34–36].

TLR8 is an important receptor protein in the innate immune pathway. Compared with other TLR members, TLR8 is more widely expressed in different subtypes of immune cells. Regulatory T cells (Treg) have strong immune response inhibition ability, which is the main obstacle to effective cancer immunotherapy. Reversing Treg inhibition is a necessary and sufficient condition leading to tumor inhibition. TLR8 is also the only protein in the TLR family that can reverse Treg-induced immunosuppression [37–39]. TLR8 activation can also enhance antibody-dependent cell-mediated cytotoxicity (ADCC) [40–42].

In 2013, the structure of an unliganded pre-formed human TLR8 homodimer was revealed (PDB code: 3W3G), together with the crystal structures of TLR8 in complex with three different agonists: CL097, CL075 (PDB codes: 3W3J, 3W3K, respectively) and resiquimod (PDB codes: 3W3L, 3 W3 M, 3W3N) [43]. The agonist ligand-induced dimers showed conformational change upon ligand binding with a dimerization interface increased surface area of 860 Å2, as both a hinge closing motion and a relative rotation of the homodimers. Ligand binding expands the gap in the top lateral face of the leucine-rich region (LRR) and results in an altered protein-protein interface [43]. Furthermore, a series of pyrido[3,2-d] pyrimidine-based toll-like receptor 7/8 dual agonists have been designed and synthesized, which exhibit potent and near-equivalent agonistic activities toward TLR7 and TLR8 with great potential as single agents or in combination with PD-1/PD-L1 blockade for cancer immunotherapy [44].

As for the ligands of the TLRs, there are three FDA-approved TLR ligands, BCG for TLR2/4, the monophosphoryl lipid A (MPLA) for TLR4, and imiquimod (IMQ) for TLR7. Several TLR ligands have been shown to have an anti-tumor effect in different types of cancer [2]. TLR ligands can be effective as monotherapy, but they are more commonly used in combination therapy as vaccine adjuvants [36]. Their efficacy as immunotherapeutic agents are primarily dependent on the induction of T-cell immunity-antigen uptake, processing, and presentation; dendritic cell maturation, and T-cell activation [2]. TLR ligands as monotherapies have varied efficiency; there are several reports showing a modest effect of TLR stimulation in clinical trials, such as the TLR9 ligand CpG-ODN in glioblastoma [45] and the TLR7 ligand 852A in hematological malignancies [46]. The mechanism is still under investigation as to why some patients responded very well to TLR ligands while others did not. One of the reasons may be the difference between TLR expression or immune infiltration within the tumor [2, 47]. Clinical trials with TLR agonists used as adjuvants have been reported [47].

## Expression and clinical significance of TLR7/TLR8 in different cancers

The expression of TLR7 and TLR8 in different kinds of cancers has been studied. TLR7 and TLR8 have a wide range of expression. More importantly, the clinical significance of TLR7 and TLR8 has been extensively explored. Here we have summarized a few of the examples.

### Pancreatic cancer

A study on the expression and prognostic value of TLRs in pancreatic cancer patients treated with neoadjuvant therapy demonstrated that both TLR7 and TLR9 predicted a favorable postoperative outcome in a separate analysis adjusted for background variables [48]. Compared to earlier stages and chronic pancreatitis, the expression of TLR7/TLR8 was increased in stage I-IV pancreatic cancer, which was upregulated in a stage-dependent manner in advanced tumors. Stimulation of TLR7/TLR8 expression resulted in elevated NF-κB and COX-2 expression and, more importantly, increased cancer cell proliferation and reduced chemosensitivity. Functional analysis in human pancreatic cancer cells points to a significant role of both TLR7 and TLR8 in chronic inflammation-mediated signaling transduction, leading to tumor cell proliferation and chemoresistance [49].

Knockout mouse studies revealed that stromal but not neoplastic TLR7 is required for the TLR7/8 agonist R848-mediated responses. In patient samples, TLR7 is ubiquitously expressed in stroma across all stages of pancreatic neoplasia, but epithelial TLR7 expression is relatively uncommon. R848 remodels tumor and host responses to promote survival in pancreatic cancer, induces anti-tumor responses and attenuates cachexia in murine models of pancreatic ductal adenocarcinoma (PDAC). R848-treated mice demonstrated a near-doubling of survival duration. As a result, R848 could be useful in the treatment of PDAC and cancer-related cachexia [50]. Another study with tumor biopsies from 154 stage I-III PDAC patients with tissue microarray slides and immunohistochemistry to assess expression of different TLRs showed strong TLR7 expression only in 14 (9%) patients. The multivariate analysis showed that patients with negative TLR7 expression had a poor prognosis [51].

### Colorectal cancer (CRC)

A study showed high tissue TLR7 expression in CRC patients with a better prognosis and lower levels of plasma C-reactive protein (CRP) [52]. However, another study of tissue microarray samples from 825 patients with CRC who underwent surgery showed that TLR7 expression exhibited no prognostic value in the survival analysis, whereas high TLR5 expression was associated with a better prognosis in CRC patients [53]. A mouse model study showed that a defect in TLR7 of pDCs is responsible for the aggravation of colitis-associated colon cancer and that TLR7 ligand can mitigate colitis-associated colon cancer [54]. In another mouse CRC model study, R848 in combination with thioridazine and loratadine significantly delayed tumor development and prolonged survival, which was associated with enhanced T-cell immune response and DC maturation [55]. In malignant tumors, myeloid-derived suppressor cells (MDSCs), one of the most dominant cellular components comprising the tumor microenvironment, play a crucial role in tumor growth, drug resistance, recurrence, and immune escape [56, 57]. Combining oxaliplatin with TLR agonists R848 reversed the functional orientation of MDSCs towards M1-like macrophages and strengthened oxaliplatin’s antitumor effect, indicating that TLR7/8 agonists have great potential as a new immunologic adjuvant in chemotherapy for oxaliplatin-resistant CRC [58]. R848, particularly when combined with anti-CD200R, improves the antitumor effects of TLR7 signaling and the local tumor microenvironment by altering the phenotype of intratumoral myeloid cells [59].

A study on TLR7 polymorphism for cetuximab-based chemotherapy in patients with metastatic CRC (mCRC) found that patients with the TLR7 rs3853839 G/G variant receiving cetuximab-based chemotherapy had a trend toward longer progression free survival (PFS) than those with any C variants, implying that TLR7 rs3853839 predicts the outcome of cetuximab-based chemotherapy in mCRC patients [60]. DSR-29133, a potent selective TLR7 agonist, can induce anti-tumor immune responses that can be further enhanced through combination with low-dose fractionated radiotherapy in different murine solid tumor models, including CRC (CT26) [61].

### Melanoma

TLR7 agonist treatment inhibited tumor-associated macrophages in B16F10 melanoma [62]. Analysis results of normalized gene expression and corresponding clinical data of patients with skin cutaneous melanoma demonstrated that TLR7 and 8 expressions correlated with the expression of immune biomarkers and positively predicted the clinical outcome of patients with melanoma [63]. Meanwhile, in a preclinical melanoma mouse model, the TLR7 agonist IMQ improves T and NK cell function during BRAF-targeted therapy [64]. The topical application of TLR7 agonist IMQ in combination with other drugs, such as ipilimumab, for patients with malignant melanoma has been reported with successful results [65, 66].

### Non-small cell lung cancer (NSCLC)

The gene expression analysis on a total of 33 advanced NSCLC patients treated with immune checkpoint inhibitors (ICI) evaluating the expression levels of 365 immune-related genes showed that high TLR7 expression levels were significantly associated with a lack of response to immunotherapy and the multivariate analysis confirmed TLR7 RNA expression as an independent predictor for both poorPFS and overall survival (OS) in advanced NSCLC patients treated with immunotherapy [67]. Furthermore, TLR7 expressed by malignant cells promotes tumor progression and metastasis through the recruitment of myeloid-derived suppressor cells in NSCLC [68]. A preclinical study showed that TLR7 agonists inhibit the growth and metastasis of lung cancer cells through immune activated mesenchymal stem cells [69]. Another study showed that R848-based stimulation of APCs in the tumor microenvironment resulted in the mobilization of an antitumor CD8^+^ immune response for treating metastatic NSCLC [70]. In addition, the TLR7/8 agonist R848 optimizes host and tumor immunity to improve therapeutic efficacy in murine lung cancer [71]. Although many studies have demonstrated that TLR7 agonists can enhance anti-tumor immune responses, these agonists also stimulate TLR7-expressing tumor cells. High TLR7 expression in the primary tumor confers poor clinical outcome and resistance to chemotherapy in lung cancer patients. This pro-tumorigenic effect of TLR7 has been validated in murine models of lung carcinoma [72].

Stomach adenocarcinoma.

A study in 30 patients with gastric cancer showed that the mRNA and protein expression levels of TLR7 were significantly downregulated in gastric cancer tissues. IMQ significantly increased TLR7 protein expression levels in SGC-7901 cells and promoted the secretion of proinflammatory cytokines such as TNF and IL-6 [73].

Overexpression of TLR7 in patients with advanced stomach adenocarcinoma (STAD) indicates a higher degree and poorer prognosis. In addition, TLR7 expression was positively correlated with immune cell infiltration and immune checkpoint expression. Therefore, TLR7 can be used as a novel diagnostic biomarker, progression and prognostic indicator, and immunotherapeutic target for stomach adenocarcinoma [74]. Conjugation of TLR7 agonist to gastric cancer antigen MG7-Ag exerts antitumor effects [75] and have synergistic antitumor effects with 5-fluorouracil via T cell activation and MDSCs inhibition [57, 76].

### Other types of cancer

A meta-analysis of the prognostic role of TLRs in cancer showed that higher expression levels of TLR7 in tumor tissues could predict poorer survival, suggesting the expression level of TLR7 in cancerous tissue may have a prognostic value in patients with various cancers [77]. TLR7 was found in 80% of human intrahepatic cholangiocarcinoma (ICC), but not in any normal human bile duct epithelium. Inhibition of TLR7 and TLR9 reduces human cholangiocarcinoma cells’ proliferation and tumor development [78]. Another study on the MDSCs isolated from breast cancer patients revealed that STAT3 inhibition and TLR7/8 pathway stimulation in MDSCs repolarize and suppress MDSCs of breast cancer patients [79]. In a study with late advanced head and neck squamous cell carcinoma (HNSCC) patients, 42.9% of the patients had an increase in TLR7 levels [80]. In another study, immunohistochemistry analysis was performed in 49 patients with human papillomavirus-positive (HPV^+^) and 28 patients with HPV^−^ patients of tongue squamous cell carcinoma. TLR7 expression did not correlate with survival in either the HPV^−^ or HPV^+^ cases [81]. Expression of TLRs in ovarian cancer results showed that advanced-stage patients with TLR7 positivity had a lower OS than patients with negative TLR7 and TLR7 can be an important prognostic marker in ovarian cancer [82]. TLRs and TLR-targeted immunotherapy against glioma [83]. While some TLR agonists exhibited survival benefit in clinical trials, TLR agonists can be used as immune modulators to enhance the efficacy of other treatment, to avoid dose accumulation. TLRs agonists can potentiate immune checkpoint delayed resistance to PD-1/PD-L1 blockade by upregulating PD-1/PD-L1 overexpression, thus unleash powerful antitumor responses when combined with immune checkpoint inhibitors [84].

A retrospective study on 150 Finnish nasopharyngeal carcinoma (NPC) tumor samples by immunohistochemistry showed that TLR7s were highly expressed in NPC and patients with positive TLR7 tumor expression had better OS than those with no TLR7 expression. TLR7 seems to be an independent prognostic factor for non-endemic NPC [85]. A study on the gene expression of different TLRs, including TLR7 and TLR8, in 63 patients with intra-epithelial neoplasia (CIN2) showed that women with CIN2 regression showed significantly higher baseline levels of TLR7 and a non-significant trend for higher TLR8 expression, suggesting the potential use of TLR-agonists for the treatment of CIN2 lesions [86]. When combining IMQ, photodynamic therapy (PDT) had a better effect on cutaneous squamous cell carcinoma (cSCC) than either IMQ or PDT alone [87]. Another preclinical study revealed that combining a CD122-preferential IL-2 pathway agonist and a TLR7/8 agonist improves systemic antitumor CD8^+^ T cell cytotoxicity [88]. A study on the expression of TLR8 and TLR7 on bladder transitional cell carcinomas (TCCs) between low-grade (LG) and high-grade (HG) and between non-muscle invasive bladder cancer (NMIBC) and muscle-invasive bladder cancer (MIBC) in 25 patients who underwent transurethral resection showed an increased expression of TLR7 in LG TCC and NMIBC, and a prevalent expression of TLR-8 in HG TCC and MIBC [89]. A retrospective study on 166 retrospective primary oral squamous cell carcinoma (OSCC) samples by immunohistochemical staining of TLR7 showed that the low expression of TLR7 in tumor and high expression of TLR7 in stroma predict a good clinical outcome for OSCC patients, and stroma fibroblast-like cells might be amenable to immunotherapy by a TLR7 agonist [90].

A recent study reported the design and synthesis of a series of pyrido[3,2-d] pyrimidine-based TLR7/8 dual agonists with potent and near-equivalent agonistic activities against TLR7 and TLR8. In vitro, TLR7/8 agonists significantly induced the secretion of IFN-α, IFN-γ, TNF-α, IL-1β, IL-12p40, and IP-10 in human peripheral blood mononuclear cell (PBMC) assays. In vivo, TLR7/8 agonists significantly suppressed tumor growth in CT26 tumor-bearing mice by remodeling the tumor microenvironment. Additionally, TLR7/8 agonists markedly improved the antitumor activity of PD-1/PD-L1 blockade and led to complete tumor regression. These results demonstrated that TLR7/8 agonists held great potential as single agents or in combination with PD-1/PD-L1 blockade for cancer immunotherapy [44].

Different TLRs play different roles in different cancers [68, 91] .Increased expression of TLR7 has also been observed in more advanced EC patients [92, 93] and correlated to the grade of differentiation in esophageal SCC [12]. More and more studies have shown that TLRs also play an important role in the occurrence and development of cancer [94, 95]. A combined TLR7/TLR9/GATA3 score can predict prognosis in biliary tract cancer [96]. High expression of TLR7, TLR9, and GATA3 was associated with improved OS. In the multivariate Cox proportional-hazards model for OS, the TLR/TLR9/GATA3 score was found to be an independent prognostic factor for OS [96].

## The TLR7/8 signaling pathway

Both TLR7 and TLR8 agonists act via the TLRs signaling pathway through canonical adapter molecule myeloid-differentiation primary response gene 88 (MyD88), as the main linker protein to mediate downstream signaling pathways [97]. As a common natural ligand, ssRNA has been identified for both TLR7 and TLR8. However, the signaling pathways are different due to different linker proteins that determine their various biological effects [98]. Upon activation of TLR7/8 ligands by TLRs in the endosomes, MyD88 binds to the cytoplasmic portion of TLRs through interaction between individual TIR domains. Receptor dimerization leads to downstream signaling, eventually leading to nuclear localization of transcription factors for pro-inflammatory factors. MyD88 links to IRAK family kinases and downstream biologic effects of these signaling pathways through NF-κB, c-Jun N-terminal kinase/activator protein 1 (AP-1), and interferon regulatory factors (IRFs). After TLR ligands stimulation, IRAK-4, IRAK-1, IRAK-2, IRAK-3, and TRAF6 are recruited to the receptor, which induces association of IRAK-1 and MyD88 via the death domains. IRAK-4 then phosphorylates IRAK-1. Phosphorylated IRAK-1 induces the activation phosphorylation of the IKK complex, consisting of IKKα, IKKβ, and NEMO/IKKγ, and IRF3/7, and thereby induces the activation of the transcription factors NF-κB and IRF3/7, respectively [97, 99–101], which results in the production and release of proinflammatory cytokines and chemokines [19, 102], such as TNFα, IL12p40, IFNα, and IFNγ [97, 99–101]. These then can inhibit apoptosis, increase inflammatory cytokine release and antimicrobial immunity, and potentially leading to autoimmune and neoplastic diseases [99, 100]. Studies in humans have shown that MyD88 mediates colorectal cancer cell proliferation, migration and invasion via NF-κB/AP-1 signaling pathway [103] and TLR4/MyD88 signaling determines the metastatic potential of breast cancer cells [95].

R848 and motolimod (VTX-2337) are second-generation experimental derivatives of IMQ, an imidazoquinoline with immunostimulatory properties originally approved by the US Food and Drug Administration for the topical treatment of actinic keratosis and genital warts more than 20 years ago. Both resiquimod and motolimod operate as agonists of TLR7 and/or TLR8, which can promote macrophage activation and induce a Th1 response through NFκB activation, thus delivering adjuvant-like signals to APCs. NFκB-TLR signaling pathway play an important role in different tumors, such as lung cancers [104]. Currently, these compounds are being investigated as immunostimulatory agents for the treatment of various malignancies, in addition to tumor vaccination and anti-virus treatment [105]. TLRs on T cells are involved in the regulation of T cell function. TLRs serve as co-stimulatory molecules and activate T cell immunity. TLR agonists are used to activate T cell-mediated antitumor responses. The activation of both innate and adaptive immune responses using TLR agonists can improve tumor therapy [97, 106].

**Fig. 2:**
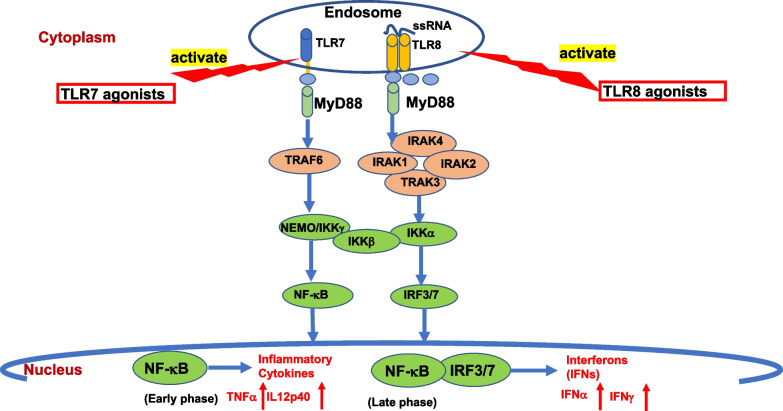
Toll-like receptors 7/8 mediated downstream signaling pathways. TLRs located on the cell surface or endosome signal through MyD88-independent pathways or MyD88-dependent pathways. Upon activation of TLR7/8 ligands by TLRs in the endosomes, MyD88 binds to the cytoplasmic portion of TLRs through interaction between individual TIR domains. Receptor dimerization leads to downstream signaling, eventually leading to nuclear localization of transcription factors for pro-inflammatory factors. After TLR ligands stimulation, IRAK-4, IRAK-1, IRAK-2, IRAK-3, and TRAF6 are recruited to the receptor, which induces association of IRAK-1 and MyD88 via the death domains. IRAK-4 then phosphorylates IRAK-1. Phosphorylated IRAK-1 induces the activation phosphorylation of the IKK complex, consisting of IKKα, IKKβ, and NEMO/IKKγ, and IRF3/7, and thereby induces the activation of the transcription factors NF-κB and IRF3/7, respectively, which induce the secretion of different inflammatory cytokines, such as TNFα, IL12p40, IFNα, and IFNγ. Abbreviations: *MyD88* (myeloid differentiation factor 88), *IRAK* (IL-1 receptor-associated kinase), *IRF* (interferon regulatory factor), *TRAF* (tumor necrosis factor receptor-associated factor), *NF-κB* (nuclear factor kappa-light-chain-enhancer of activated B cells)

TLRs are a pathogen sensor family that recognizes bacterial and viral ligands and activates innate immune sensing [107]. TLR activation polarizes macrophages to become pro-inflammatory [107]. Recently, a study reported that activation of TLR7 and TLR9 increases the phagocytosis of monocyte-derived macrophages and causes anemia and thrombocytopenia associated with inflammation and infection [108]. Researchers have used different TLR ligands in various cancer models to analyze their activities during the transformation of tumor-associated macrophages (TAMs) into tumor-killing macrophages [107]. The TLR7/8 agonists activation through MyD88 signaling pathway is illustrated in fig. 2 (Fig. 2).

The agonists of TLR7 directly activated purified pDCs and monocytes, whereas TLR8 agonists directly activated purified myeloid DCs, monocytes, and monocyte-derived dendritic cells (mDCs). Accordingly, TLR7-selective agonists in inducing IFN-alpha and IFN-regulated chemokines were more effective than TLR8-selective agonists. Meanwhile, TLR8 agonists were more effective than TLR7 agonists in inducing proinflammatory cytokines and chemokines, such as TNF-α, IL-12, and MIP-1α [109].

A TLR7/8 bi-specific agonist can significantly enhance cytokine secretion. TLR7/8 agonist nanoparticles triggered DC activation and expansion, leading to expansion of antigen specific CD8^+^ T cells and enhanced CTL response, which resulted in significant prophylactic and therapeutic efficacy in melanoma, bladder, and renal cell carcinoma tumor models [110]. A recent study showed that imidazoquinoline-based TLR7/8 agonist (522) can enhance the anti-cancer efficacy of monoclonal antibodies with improved ADCC in vitro and in vivo by inducing robust pro-inflammatory cytokine secretion and activating NK cells [42].

R848, which usually triggers TLR7 and TLR8 on dendritic cells, macrophages, and neutrophil cells, activates CD56^bright^CD16^−^ NK-cell subset only via TLR8. Indeed, specific TLR8 but not TLR7 agonists increased cytokine production and cytotoxic activity of CD56^bright^CD16^−^ NK cells. Importantly, these activities were also observed in peritoneal NK cells from patients with metastatic ovarian carcinoma, predominantly in the CD56^bright^CD16^−^ subset [111]. In a preclinical study, R848 exhibited a robust antitumoral effect in mouse breast cancer models by reducing tumor vasculature and inducing tumor cell apoptosis. In addition, R848 activated CD4^+^ T cells in the peripheral blood. A synergistic antitumoral effect of R848 and the antiangiogenic agent, sunitinib, was observed, suggesting that the TLR7/8 agonist may be a potential adjuvant to potentiate the effect of antiangiogenic therapy [112]. R848 can activate the NK cells and result in accessory cell-dependent IFN-γ production [113]. A recent study on the therapeutic applications of TLR agonists in acute myeloid leukemia (AML) demonstrated that R848 has direct antileukemic effects and considerably impairs the growth of human AML cells in immunodeficient mice, suggesting that treatment with TLR8 agonists may be a promising new therapeutic strategy for AML [114].

The mechanism of R848’s efficacy in cancer immunotherapy is not completely clear. R848 optimizes host and tumor immunity to improve therapeutic efficacy in murine lung cancer. Treatment with R848 is effective in various types of cancer, such as breast, pancreatic, and colorectal cancer [112, 115, 116]. Moreover, recent studies focusing on macrophages have highlighted the therapeutic efficacy of R848, either alone or in combination with other drugs, in lung cancer [117, 118].

IRMs with a TLR-8 agonist component (3 M-002 and 3 M-003) can modulate NK-cell function both in vitro and in vivo*,* and those distinct indirect pathways control human NK-cell activation by TLR-7 and TLR-8 agonists. IL-18 and IL-12p70 were primarily required for induction of IFN-γ by both synthetic and natural TLR-8 ligands, while type I IFN was required for induction of CD69 on NK cells by the TLR-7 agonist 3 M-001 [119]. Recently, advances in technology and applications of nanoimmunotherapy for cancer provides a unique paradigm to simultaneously tackle challenges, including effective “targeted” delivery of tumor antigens, sustained ICD mediation, and “cold” tumor microenvironment remodeling [120, 121]. TLR7/8 agonist-loaded nanoparticles augment NK cell-mediated antibody-based cancer immunotherapy by promoting NK cell activation [122].

TLR7/8 agonists in clinical trials for therapeutic treatment have been reported in different clinical applications [97]. Although many of the TLR7/8 agonists applied in immune-mediated diseases were focused on viral infection, such as hepatitis B virus (HBV) or HIV, vaccines and adjuvant, such as IMQ for influenza vaccine [123] and HBV vaccine [124], more and more TLR7/8 agonists have been tried in cancer immunotherapy [8, 97, 106] and gene-modified adoptive cell therapies, including TLR signaling domains in CAR T-cells [6]. There is accumulating evidence suggesting that TLR signals play an important role in the regulation of hematopoietic stem/progenitor cells (HSPCs). TLR7/8 agonist treatment increases bone marrow resident DCs as well as hematopoietic progenitor expansion and mobilization [125].

### TLR7 agonists

SZU-106, a small-molecule TLR7 agonist, linked to the cell surface with a pegylated linker to produce a novel whole-tumor-cell vaccine, showed immune-activating responses in both in vitro and in vivo experiments, with a great potential to treat advanced malignant tumors [126]. Another TLR7 agonist called SZU-101 demonstrated the systemic antitumor effect on a murine model of T cell lymphoma in vivo. The novel combination of intratumourally administered SZU-101 and doxorubicin generated strong cytokine production and enhanced the cytotoxic T lymphocyte response, leading to the eradication of both local and distant tumors in tumor-bearing mice [127].

GS-9620 is a potent oral TLR7 agonist, which can induce the secretion of cytokines and chemokines such as IFN-α, upregulate the expression of TLR7 and interferon-stimulated genes (ISGs) in PBMCs and liver tissue, activate NK-cells and T-cell subpopulations, and initiate an anti-viral immune response, significantly reducing serum levels of HBV DNA and expression of viral antigens [97, 128, 129]. In a preclinical study, GS-9620 induced durable antiviral efficacy in woodchucks chronically infected with woodchuck hepatitis virus [130] and suppression of hepatitis B virus [97] in different animal models [97]. The TLR7 agonist 852A has been studied in a phase II trial in patients with recurrent malignant hematologic tumors. It can promote the production of various cytokines, pDC maturity, and the killing of tumor cells by recruiting pDCs to tumor tissues [46].

### TLR8 agonists

TLR8 is an intracellular type I transmembrane protein, which is mainly expressed in myeloid cells such as monocytes, macrophages, and neutrophils in the human body [32, 131]. TLR8 can reverse the immunosuppressive function of Treg cells and has a strong anti-tumor effect. At the same time, the activation of TLR8 can also induce the apoptosis of MDSCs that inhibit immune response, leading to the activation and enhancement of tumor immune response [132]. Tumor Treg and MDSC are the main reasons for the failure of tumor immunotherapy. Therefore, reversing Treg and MDSC-mediated immunosuppression by activating TLR8 can trigger a strong immune response and produce a strong anti-tumor effect [79, 132].

TLR8 is located in the endosomal compartment of monocytes and mDCs, and its activation stimulates the release of inflammatory mediators, including Th1-polarized cytokines [133, 134]. Personalized DC vaccine-induced T cell immunity, which targets tumor-specific antigens, has been demonstrated to be a promising form of tumor immunotherapy in patients with melanoma [135].

TLR8 agonists stimulate NK cell activity, enhance antibody-dependent cytotoxicity, and induce the production of IFN-γ. TLR8 signaling mainly results in NF-kB pathway activation and subsequent proinflammatory cytokines and chemokines expression [136].

Compared with TLR7 and TLR9, TLR8 is more widely distributed in immune cell subsets, and selective agonists can effectively activate mDC and monocytes. TLR8 activated mDC is very suitable for producing an acquired immune response against tumor cells. Activated mDCs phagocytize apoptotic and necrotic tumor cells and effectively cross-present tumor-associated antigens to CD8^+^ CTL [137]. Since the involvement of TLR8 promotes the maturation of mDC and promotes the development of innate and acquired anti-tumor immune responses, the addition of TLR8 agonists to some standard cancer anticancer drugs, such as anthracycline chemotherapy, monoclonal antibody (mAb) therapy or radiotherapy, may significantly enhance the anti-tumor response. In particular, enhancing tumor cell killing through ADCC may be an important therapeutic opportunity for TLR8 specific agonists [137]. TLR8 ligand-activated monocytes potently co-stimulate early γδ T-cell activation but fail to provide accessory cell function for in vitro expansion of γδ T cells [138].

Tumor immunotherapy has encountered a common problem with drug resistance. The lack of efficacy is often attributed to the “cold” immune status of tumors, with a low number of tumor-infiltrating lymphocytes (TILs) but a high number of suppressive immune cells, including TAMs, MDSCs, or Tregs [139–143]. TLRs play central roles in the initiation of innate immune response, and also control adaptive immunity activation by enhancing peripheral and tumor-infiltrating CD8^+^ T cell cytotoxicity in patients with gastric cancer, therefore play an important immunomodulatory activity [144]. Intratumor heterogeneity, especially the deficient mismatch repair /microsatellite instability status is used as a biomarker in clinical practice to predict favorable response to immunotherapy and prognosis [145]. The T-cell receptor (TCR) repertoire diversity of CD4^+^ TILs were significantly higher than those of CD8^+^ TILs. Intratumor heterogeneity of CD4/CD8 T-cell ratio and CD8^+^ TIL repertoire across center regions was lower than that across margin regions. The amount and TCR repertoire ITH of CD4^+^ and CD8^+^ TILs and mean clonality of CD8^+^ TILs in tumor centers were associated with relapse in lung adenocarcinoma patients, suggesting potential clinical significance of TCR repertoire [146]. The complex architecture inside tumors may further complicate the intratumor TCR heterogeneity [147]. The heterogeneity of TCR repertoire has also been used to predict distant metastasis after treatment and indicate prognosis [148–150]. Animal model showed that superantigen mutant induced tumor-targeting and T lymphocyte infiltrating in cancer immunotherapy [151].

Innate immunity plays a key role in activating tumors with immunosuppression and transforming “cold” tumors without immune response into “hot” tumors with immune response. Therefore, activating innate immunity provides a new direction for tumor immunotherapy. TLR8 agonists can lead to the release of various proinflammatory cytokines, such as IL-6, IL-12, TNF-α, and IFN-γ and activate the intracellular signal transduction pathway and activate innate immunity [152, 153], which plays a bridge role between innate immunity and adaptive immunity [153]. Repolarizing TAMs could be a promising strategy to reshape the tumor immune microenvironment and promote antitumor activity when combined with immune checkpoint blockades (ICBs) [154]. TLR7 and 8 agonists, such as IMQ and resiquimod, are potent immunostimulatory molecules with the potential to repolarize macrophages [139]. Combination strategies such as ICB and adoptive cell therapy, innate immune modifiers and cancer vaccines, as well as combination therapies with other therapeutic modalities can maximize the benefits of cancer immunotherapy [155]. According to the action mechanism, TLR8 agonists are effective not only as monotherapy but also as combinations with other drugs, including immune checkpoint inhibitors, such as PD-1, PD-L1 monoclonal antibodies (mAbs) and chemotherapy drugs, to further expand the indications and enhance the efficacy [139, 152, 156].

Recent studies on a highly potent and specific inhibitor of TLR8 (TH1027) and its X-ray crystal structure in complex with TLR8 demonstrated that it can prevent TLR8 from activation and therefore suppress TLR8-mediated inflammatory responses in human monocyte cell lines, PBMCs, and rheumatoid arthritis patient specimens, implying a strong therapeutic potential against autoimmune diseases [22].

Although there were many studies showing TLR agonists as immunotherapeutic agents with anti-tumor activities, other studies revealed TLR agonists with tumor progression with production of immunosuppressive cytokines, increased cell proliferation, and resistance to apoptosis [2]. Therefore, it seems that TLR signaling can act as a double-edged sword in cancer, with its pro- and anti-cancer roles that have been reviewed by others [91, 157, 158]. The anti-tumoral effects can result from direct induction of tumor cell death and/or activation of efficient anti-tumoral immune responses, and the pro-tumoral effects may be due to inducing tumor cell survival and proliferation or by acting on suppressive or inflammatory immune cells in the tumor microenvironment [91]. For example, one study showed TLR7 stimulation induced tumor cell survival and resistance to chemotherapy [91]. In the lung tumor mouse models, injection of TLR7 agonists increased intratumoral infiltration by MDSCs which could indirectly contribute to the pro-tumoral effects of TLR7 stimulation of tumor cells [159]. In addition, TLR7 expressed by tumor cells play roles in promoting tumor progression in both mouse and humans [49, 160]. However, the pro-tumoral effects were only reports with TLR1/2, TLR4, YLR7 an TLR9, no pro-tumoral effect has been reported yet [91]. The therapeutic approach with TLR agonist is trying to stimulate the TLR expressing immune cells to improve anti-tumor immune response. However, since some tumor cells also express TLR, their stimulation may lead to increased tumor cell survival and proliferation, or drug resistance to chemotherapy. Different TLR agonists have been developed for the potential therapy in cancer immunotherapy, with some already in TLR-related clinical trials [99]. When using TLR agonists, TLR expression by tumor cells should be considered to avoid the TLR pro-tumoral effect in cancers.

## TLR7/8 agonists related clinical trials

At present, no TLR8 selective agonists have been approved for clinical applications yet. Motolimod/VTX-2337 (NCT00688415, NCT01836029, NCT01666444), GS9688.

(NCT03615066, NCT03491553), and DN1508052–01 (NCT03934359) are TLR8 agonists in clinical trials with different clinical implications, including various cancers and chronic hepatitis B (Table 1). Many clinical trials attempt to enhance the anti-tumor effect by using agonists to cover both TLR7 and TLR8 targets. However, more clinical trials targeting TLR8 selectively are attracting attention.

**Table 1:** TLR7 and 8 agonists in clinical trials

Agonist	Targeted TLR	NCT#	Phase	Application	Sponsor	Mechanism	Status	Enrollment
852A	TLR7	NCT00319748	II	Breast, ovarian, endometrial and cervical cancers	Masonic Cancer Center, University of Minnesota	Anti-tumor	Completed	15
BNT411	TLR7	NCT04101357	I	Solid tumors, ES-SCLC	BioNTech Small Molecules GmbH	Anti-tumor	Recruiting	60
DN1508052	TLR8	NCT03934359	Ib/II	Advanced Solid Tumors	Shanghai De Novo Pharmatech Co., Ltd.	Anti-tumor	Unknown	25
DSP-0509	TLR7	NCT03416335	I/II	Advanced solid tumors	Sumitomo Pharma Oncology, Inc.	Anti-tumor	Recruiting	100
Immiquimod	TLR7	NCT00899574	II	Breast cancer	NYU Langone Health	Anti-tumor	Completed	10
Immiquimod	TLR7	NCT01421017	I/II	Breast cancer	NYU Langone Health	Anti-tumor	Completed	31
Immiquimod	TLR7	NCT04116320	I	Advanced solid tumors	Craig L Slingluff, Jr	Anti-tumor	Recruiting	32
MEDI9197 (3 M-052)	TLR7/8	NCT02556463	I	Solid tumors or CTCL	MedImmune LLC	Anti-tumor	Terminated	53
TLR agonist PolyICLC	TLR7/8	NCT02643303	I/II	Advanced solid tumors	MedImmune LLC	Anti-tumor	Completed	58
VTX-2337	TLR8	NCT01289210	I, II	Low-grade B-cell lymphomas	Celgene	Anti-tumor	Terminated	2
VTX-2337	TLR8	NCT01294293	I	Ovarian epithelial, fallopian tube, or peritoneal cavity cancer	Celgene	Anti-tumor	Completed	20
VTX-2337	TLR8	NCT01334177	I	Squamous Cell Carcinomas of the Head and Neck (SCCHN)	Celgene	Anti-tumor	Completed	13
VTX-2337	TLR8	NCT01666444	II	Epithelial ovarian, fallopian tube or primary peritoneal cancer	Celgene	Anti-tumor	Completed	297
VTX-2337	TLR8	NCT01836029	II	Squamous cell carcinoma of the head and neck	Celgene	Anti-tumor	Completed	195
VTX-2337	TLR8	NCT02431559	I,II	Ovarian cancer	Celgene	Anti-tumor	Completed	53
VTX-2337	TLR8	NCT02650635	Ib	Advanced solid tumors	Celgene	Anti-tumor	terminated	4
VTX-2337	TLR8	NCT03906526	Ib	Head and neck cancer	Celgene	Anti-tumor	Completed	15
GS-9620	TLR7	NCT02166047	II	Chronic hepatitis B virus in virally suppressed subjects	Gilead Sciences	Antiviral treatment	Completed	162
GS-9688	TLR8	NCT03491553	II	Virally suppressed adults with chronic hepatitis B	Gilead Sciences	Antiviral treatment	Completed	48
GS-9688	TLR8	NCT03615066	II	Naive CHB patients	Gilead Sciences	Antiviral treatment	Completed	67
imiquimod	TLR7	NCT00453050	I	Melanoma	3 M	Enhancing vaccination	Completed	11
PolyICLC	TLR7/8	NCT04364230	I/II	Melanoma	Celldex Therapeutics	Enhancing vaccination	Recruiting	44
Resiquimod	TLR7/8	NCT02126579	I/II	Melanoma vaccination	Mologen AG	Enhancing vaccination	Active, not recruiting	62
Resiquimod	TLR7/8	NCT00960752	II	Melanoma	Mologen AG	Enhancing vaccination	Completed	47
Resiquimod	TLR7/8	NCT02126579	I/II	Melanoma vaccination	Mologen AG	Enhancing vaccination	Active, not recruiting	62
TLR agonist PolyICLC	TLR7/8	NCT01585350	I	Melanoma vaccination	Oncovir, Inc.	Enhancing vaccination	Completed	52

The TLR7 agonist 852A phase-II study revealed anti-tumor activities in patients with breast, ovarian, endometrial, and cervical cancers [161]. Another study of 852A in patients with refractory metastatic melanoma revealed 2/3 patients who completed 24 weeks of therapy exhibited stable status without tumor progression, and no adverse events occurred [97, 162]. *Brenda J. Weigel* et al. performed a phase II clinical study of the TLR7 agonist 852A in R/R hematological cancer patients [46]. The study included six patients with AML, five with acute lymphocytic leukemia (ALL), four with non-Hodgkin lymphoma (NHL), one with Hodgkin lymphoma (HL), and one with multiple myeloma (MM). Of the 17 cases, 13 patients completed all 24 cycles of 852A injections. Grade 3/4 toxicities included dyspnea, myalgia, nausea, malaise, fever, and cough. Patients with clinical responses included one ALL and one AML. However, nine patients showed progressive disease [46].

VTX-2337 (Motolimod), a selective TLR8 agonist developed by Ventirx, stimulates IFNγ production from NK cells and increases the cytotoxicity of NK cells against K562 and ADCC by rituximab and trastuzumab. The effects of VTX-2337 on NK cells were, in part, from direct activation as increased IFNγ production and cytotoxic activity were seen with purified NK cells. Finally, VTX-2337 augments ADCC by rituximab in PBMCs with different FcγR3A genotypes (V/V, V/F, and F/F at position 158) [133].

There were 9 trials of VTX-2337 registered in the clinicaltrials.gov database. Two of them (NCT01289210, NCT02650635) were terminated due to the slow enrollment of patients with low-grade B-cell lymphomas and R/R solid tumors treated with VTX-2337 combined with radiotherapy. The other 7 completed trials included 3 in patients with HNSCC (NCT03906526, NCT01334177, and NCT01836029), 3 in patients with ovarian cancer (NCT01666444, NCT01294293 and NCT02431559), and 1 in patients with advanced solid tumors (NCT00688415).

The preclinical research revealed that TLR8 expression was associated with CD8^+^ T cell infiltration and favorable survival outcomes. The anti-tumor effects of VTX-2337 plus cetuximab were accompanied by increased splenic lymphoid DCs, IFNγ^+^ CD4^+^ and tumor-specific CD8^+^ T cells. Patients with high TLR8 expression may get more benefit from this combination regimen than patients with low TLR8 expression [40]. A phase Ib trial (NCT01836029) of VTX-2337 combined with cetuximab in patients with recurrent or metastatic HNSCC showed that there was no protocol-defined dose-limiting toxicities, drug-related deaths, or evidence of synergistic toxicities between VTX-2337 and cetuximab. Two of thirteen patients (15%) achieved partial responses and five patients had disease stabilization equating to a disease control rate of 54%. There was a significant increase in plasma cytokines and in the frequency and activation of circulating NK cells [163]. Another study showed late-stage cancer patients remain highly responsive to immune activation by VTX-2337. Multiple biomarkers, including IL6, G-CSF, MCP-1, and MIP1-β, increased in plasma as VTX-2337 dose increased. Tumor burden, advanced age, and prior treatment history with cytotoxic agents did not moderate or modify the response predicted by nonclinical studies and confirmed in healthy volunteers [134].

In another phase Ib study in patients with HNSCC, adding VTX-2337 to cetuximab therapy resulted in enhanced T-lymphocyte stimulation and anti-EGFR-specific priming. TLR8 stimulation skewed monocytes toward M1-phenotype and reversed MDSC suppression of T-cell proliferation in vitro. VTX-2337 plus cetuximab also decreased induction of Treg and reduced markers of suppression, including CTLA-4, CD73, and membrane-bound TGFβ. Significantly increased circulating EGFR-specific T cells were observed, concomitant with enhanced CD8^+^ T-cell infiltration into tumors. The TCR clonality was increased, the costimulatory receptor CD27 was upregulated, and the inhibitory receptor T-cell immunoreceptor with Ig and ITIM domains (TIGIT) was downregulated [164].

In the Active8 randomized clinical trial, adding VTX-2337 to standard combination chemotherapy and cetuximab treatment of 195 patients with HNSCC did not improve PFS or OS, with the median PFS of 6.1 vs 5.9 months, and the median OS of 13.5 vs 11.3 months for VTX-2337 vs placebo. However, the PFS and OS of the prespecified HPV-positive subgroup were significantly longer in the VTX-2337 group than that in the placebo group, (7.8 vs 5.9 months) and OS (15.2 vs 12.6 months), respectively. In an exploratory analysis, patients with injection site reactions had longer PFS and OS (median PFS, 7.1 vs 5.9 months; and median OS, 18.7 vs 12.6 months). Significant benefit was observed in HPV-positive patients and those with injection site reactions, suggesting that TLR8 stimulation may benefit subset- and biomarker-selected patients [165]. More exploratory combination selection and subgroup definition with special biomarkers such as HPV are needed to validate the benefit of TLR8 agonists in the treatment of HNSCC.

Variable expression of TLR8 was seen in the benign and malignant epithelium of ovarian cancer patients, while expression of TLR7 was weak [166]. In the integrative development of VTX-2337 for ovarian cancer chemoimmunotherapy in a phase Ib trial (NCT01294293), the results revealed the combination produced no dose-limiting toxicities in patients with ovarian cancer. Two subjects (15%) had complete responses and seven subjects (53%) had disease stabilization [167]. In a phase 2, randomized, double-blind, placebo-controlled study of chemo-immunotherapy combination using VTX-2337 with pegylated liposomal doxorubicin (PLD) in recurrent or persistent ovarian cancer, addition of VTX-2337 to PLD did not significantly improve OS or PFS. However, in pre-specified subgroup analyses, VTX-2337-treated patients who experienced injection site reactions (ISR) had a lower risk of death compared with those who did not experience ISR. Additionally, pre-treatment in vitro responses of immune biomarkers to TLR8 stimulation predicted OS outcomes in patients receiving VTX-2337 [168]. However, there are no approved clinical application regarding VTX-2337. Although TLR8 agonists have 50 times higher potent agonistic activity than that of TLR7, the clinical dosage of VTX-2337 is relatively low, and the curative effect seems to be limited, indicating that the therapeutic index of VTX-2337 is narrow [133]. This probably explains why there are no phase III clinical trials ongoing in patients with tumors.

DN1508052 is a selective TLR8 agonist under development with a structure similar to VTX-2337. Currently, there is an open-label, multicenter phase Ib/II clinical trial ongoing both in the US (NCT03934359) and China (CTR20201728). This study is to evaluate the safety, tolerability, pharmacokinetics(PK)/pharmacodynamics(PD) and preliminary efficacy of DN1508052 as monotherapy and in combination with anti-PD-1 mAbs (Toripalimab) in patients with advanced solid tumors after standard treatment or without standard treatment. This trial covered different types of solid tumors, such as HNSCC, adenocarcinoma of stomach or gastroesophageal junction, NSCLC, SCLC, cervical cancer and triple-negative breast cancer.

As early as the 1960s, *Vassal* et al. reported improved OS in pediatric leukemia patients using the TLR agonist Bacille Calmette-Guérin vaccine, which is currently used for treating bladder cancer patients [169]. The majority of clinical trials using TLR agonists to treat hematological malignancies have focused on TLR3, TLR7/8, and TLR9. Recent investigations have shown that the synchronous application of different TLR agonists may be useful for patients with various TLRs expressed in tumors.

TLR8 plays an important role in controlling chronic viral infections. However, the role of TLR8 in chronic HBV infection is poorly understood. Compared with healthy controls, TLR8 expression and IFN-γ, TNF-α, and IL-12 induction were reduced in PBMCs from chronic hepatitis B (CHB) patients. Analysis of the temporal dynamics revealed that patients who achieved a complete response had a significantly higher level of TLR8 mRNA than those who did not, beginning at week 12 of peg-IFN-α-2a therapy [170]. The TLR8 agonist GS-9688 (selgantolimod) was discovered as a potent and selective oral TLR8 agonist for the treatment of chronic hepatitis B. In a preclinical study, a reduction in viral markers was observed in HBV-infected primary human hepatocytes treated with GS-9688 [171]. Targeting follicular helper T cells (TFH) through TLR8 signaling can improve HBsAg specific B cell responses in patients with chronic hepatitis B. TLR8 agonism can enhance HBV-specific B cell responses in CHB patients by improving monocyte-mediated TFH function and may play a role in achieving HBV functional cure [172]. The study on the safety, PK, and PD demonstrated that GS-9688 was safe and well-tolerated in virally suppressed and viremic patients with CHB and elicited cytokine responses consistent with target engagement [173]. Further studies with longer durations of GS-9688 treatment are required to evaluate efficacy.

Antiviral activity of GS-9688 has previously been evaluated in vitro in HBV-infected hepatocytes and in vivo in the woodchuck model of CHB [174]. A phase Ia trial showed oral administration of GS-9688 induces multiple immune cell responses in humans. Myeloid cell activation was evident by the upregulated expression of co-stimulatory molecules CD40 and CD86, accompanied by the production of IL-6 and IL-18 from these cells. Concomitantly, there was induction of the early activation marker CD69 on innate and adaptive lymphoid cells and enhanced expression of the effector molecules granzyme B and perforin [175]. Furthermore, the phase Ia study also showed that the single doses of up to 5 mg GS-9688 were safe and induced dose-dependent pharmacodynamics responses [176]. As a result, GS-9688 has the potential to treat chronic hepatitis B by remodeling antiviral and regulatory mediators [177]. There were 2 completed clinical trials regarding GS-9688 from the clinicaltrials.gov database (NCT03615066 and NCT03491553) in patients with chronic hepatitis B. The phase II trial results showed that IL-12p40 and IL-1ra increased in most patients treated with GS-9688 4 hours after administration.

CB06–036 is a small molecule, oral selective TLR8 agonist under development with a structure similar to GS-9688. Preclinical studies have shown that CB06–036 can induce cytokines in PBMCs that activate antiviral effector functions by multiple immune mediators with good liver targeting activity. Currently, there is a phase I clinical trial ongoing, which is a randomized, double-blind, and placebo-controlled study, to evaluate the safety, pharmacokinetics, and pharmacodynamics of single escalating doses of CB06–036 in patients with CHB [178] .

HRS9950 is another TLR8 agonist currently in phase I clinical trial in patients with CHB (NCT04464733). This phase I study was divided into three parts: the first part was to evaluate the safety, tolerability, PK, and PD of single-dose and multi-dose HRS9950 in healthy donors. The second part is to evaluate the food effect of HRS9950 on healthy donors. The third part is to evaluate the safety, tolerability, PK, and PD of multi-dose HRS9950 in patients with CHB.

## Conclusion and prospective

As innate immunotherapeutic agents, TLR7/8 agonists have the potential to induce immuno-regulation function, which is supposed to bridge the innate cellular immune to tumor immunotherapy. Although the present results demonstrated that TLR7/8 agonists alone or as an adjuvant can retard tumor growth, other studies have suggested that TLR7 is associated with carcinogenesis and immunosuppression. While many studies have been conducted on different TLR7/8 agonists, a breakthrough is urgently needed regarding the efficacy of cancer immunotherapy. Thus, the molecular mechanism of action should be further investigated in future studies. Furthermore, TLR7/8 agonists may be a potential adjuvant for future anti-angiogenic therapy. Recently, clinical research focus has expanded from the combination of different TLR target agonists to selective single TLR agonist and in selective combination with other immune target therapy regimens, such as PD-1/PD-L1, CTLA-4, TIGIT and LAG3 [179–182]. So far, there has been no breakthrough in the research of innate immune drugs regarding TLRs, and the selectivity and effectiveness are not clear yet. Emerging immunotherapies were proposed to overcome the primary and secondary resistance to existing immune checkpoint inhibitors, activate effector cells, and target immunosuppressive mechanisms in tumor microenvironment [141, 183–185]. More research needs to be explored regarding the mechanisms of the innate immune therapeutic agents. Autophagy pathway to inflammasome activation may influence the outcome of pro-tumor or anti-tumor responses depending on the cancer types, microenvironment, and the disease stage. Targeting macrophage approaches for either autophagy or inflammasome may be potential as anti-cancer strategies [186, 187].

Immunotherapy targeting inhibitory molecules like anti-CTLA-4 and anti-PD-1/PD-L1 were developed to overcome the immunosuppressive effects. These agents have demonstrated remarkable, durable responses in a small subset of patients [84]. TLRs agonists in combination of these inhibitory molecules and focus on the microenvironment and metabolic characteristics of tumor cells as well as the regulatory immune cells should be further explored [188]. Although no drug targeting TLR7/8 has been approved for cancer immunotherapy clinical application worldwide, but great efforts of global research and development have been made by focusing on TLR7/8-targeted agents for cancer immunotherapy and other types of diseases, such as patients with chronic hepatitis B. TLR agonists is considered a promising therapeutic strategy in the immunotherapy of solid tumors [189].

Clinical applications with a special subclass of patients may need to be categorized with either TLR agonist alone or agonist in combination with ICBs to specifically target that special population for cancer target immunotherapy, before it could be applied to a broad range of clinical applications. With the optimal antitumor immunity for robust enhancement of the effector T-cell response induced by tumor antigenic peptides and control or elimination of Treg cell-suppressive function, the combination of immune check point inhibitors with TLR agonists, in particular, the TLR8 agonist, may greatly improve the therapeutic potential of cancer immunotherapy. Furthermore, more novel targeting agents also need to be explored. We believe that with the continuous progress of research, the new TLR7/8 targeted drugs will improve the treatment efficiency and survival rate of patients with malignant tumors and benefit more patients.

## Data Availability

All clinical trials related information was obtained from public databases.

## Abbreviations

ADCC: Antibody-dependent cell-mediated cytotoxicity
ALL: Acute lymphocytic leukemia
AML: Acute myeloid leukemia
APCs: Antigen-presenting cells
CHB: Chronic hepatitis B
CIN2: Intra-epithelial neoplasia
CpG: Cytosine phosphate guanosine
CRC: Colorectal cancer
CRP: C-reactive protein
cSCC: Cutaneous squamous cell carcinoma
dsDNA: Double-stranded DNA
ECD: Extracellular domain
HBV: Hepatitis B virus
HG: High-grade
HIV: Human immunodeficiency virus
HL: Hodgkin lymphoma
HPSCs: Hematopoietic stem/progenitor cells
HPV: Human papillomavirus
ICBs: Immune checkpoint blockades
ICC: Intrahepatic cholangiocarcinoma
ICI: Immune checkpoint inhibitors
IFN: Interferon
IMQ: Imiquimod
IRAK: IL-1 receptor-associated kinase
IRF: Interferon regulatory factor
IRFs: Interference regulatory factors
LAG3: Lymphocyte activating gene 3
LG: Low-grade
LPS: Lipopolysaccharide
LRR: Leucine-rich region
mAbs: Monoclonal antibody
mDCs: Monocyte-derived dendritic cells
MDSCs: Myeloid-derived suppressor cells
MIBC: Muscle-invasive bladder cancer
MM: Multiple myeloma
MPLA: Monophosphoryl lipid A
MyD88: Myeloid differentiation factor 88
NETs: Neutrophils to form extracellular traps
NF-kB: Nuclear factor kappa-light-chain-enhancer of activated B cells
NHL: Non-Hodgkin lymphoma
NMIBC: Non-muscle invasive bladder cancer
NPC: Nasopharyngeal carcinoma
NSCLC: Non-small cell lung cancer
ODNs: Oligodeoxynucleotides
OS: Overall survival
OSCC: Oral squamous cell carcinoma
PAMPs: Pathogen-associated molecular patterns
PD: Pharmacokinetics
PDAC: Pancreatic ductal adenocarcinoma
pDCs: Plasmacytoid dendritic cells
PDT: Photodynamic therapy
PFS: Progression free survival
PK: Pharmacokinetics
PLD: Pegylated liposomal doxorubicin
ssDNA: Single-stranded DNA
STAD: Stomach adenocarcinoma
TAMs: Tumor-associated macrophages
TCCs: Transitional cell carcinomas
TCR: T-cell receptor
TFH: Targeting follicular helper T cells
TIGIT: T-cell immunoreceptor with Ig and ITIM domains
TIL: Tumor infiltrating lymphocytes
TIR: Toll-interleukin 1 receptor
TLR: Toll-like receptors
TMD: Transmembrane domain
TRAF: Tumor necrosis factor receptor-associated factor
Treg: Regulatory T cells
