# Correction: Targeting toll-like receptor 7/8 for immunotherapy: recent advances and prospectives

**DOI:** 10.1186/s40364-022-00445-6

**Published:** 2022-12-21

**Authors:** Hao Sun, Yingmei Li, Peng Zhang, Haizhou Xing, Song Zhao, Yongping Song, Dingming Wan, Jifeng Yu

**Affiliations:** 1 Department of Radiotherapy, the First Affiliated Hospital of Zhengzhou University, Zhengzhou, 450052 Henan China; 2 Department of Hematology, the First Affiliated Hospital of Zhengzhou University, Zhengzhou, 450052 Henan China; 3 Department of Thoracic Surgery, the First Affiliated Hospital of Zhengzhou University, Zhengzhou, 450052 Henan China; 4 Henan International Joint Laboratory of Nuclear Protein Gene Regulation, Henan University College of Medicine, Kaifeng, 475004 Henan China

**Correction: Biomark Res 10, 89 (2022)**

**https://doi.org/10.1186/s40364-022-00436-7**

The original article [1] contains an error in the following sentence:

‘When combining IMQ, photodynamic therapy (PDT) had a better effect on cutaneous squamous cell carcinoma (cSCC) than either IMQ or PDT alone.’

The mention of IMQ is erroneous and should instead be IMA (imatinib mesylate).
